# Does vimentin help to delineate the so-called 'basal type breast cancer'?

**DOI:** 10.1186/1756-9966-28-118

**Published:** 2009-08-20

**Authors:** Renata U Kusinska, Radzislaw Kordek, Elzbieta Pluciennik, Andrzej K Bednarek, Janusz H Piekarski, Piotr Potemski

**Affiliations:** 1 Department of Pathology, Medical University of Lodz, Copernicus Memorial Hospital, 4 Paderewski St., 93-509, Lodz, Poland; 2 Department of Chemotherapy, Medical University of Lodz, Copernicus Memorial Hospital, 4 Paderewski St., 93-509 Lodz, Poland; 3 Department of Molecular Cancerogenesis, Medical University of Lodz, 6/8 Mazowiecka St., 92-215 Lodz, Poland; 4 Department of Surgical Oncology, Medical University of Lodz, Copernicus Memorial Hospital, 4 Paderewski St., 93-509, Lodz, Poland

## Abstract

**Background:**

Vimentin is one of the cytoplasmic intermediate filament proteins which are the major component of the cytoskeleton. In our study we checked the usefulness of vimentin expression in identifying cases of breast cancer with poorer prognosis, by adding vimentin to the immunopanel consisting of basal type cytokeratins, estrogen, progesterone, and HER2 receptors.

**Methods:**

179 tissue specimens of invasive operable ductal breast cancer were assessed by the use of immunohistochemistry. The median follow-up period for censored cases was 90 months.

**Results:**

38 cases (21.2%) were identified as being vimentin-positive. Vimentin-positive tumours affected younger women (p = 0.024), usually lacked estrogen and progesterone receptor (p < 0.001), more often expressed basal cytokeratins (<0.001), and were high-grade cancers (p < 0.001). Survival analysis showed that vimentin did not help to delineate basal type phenotype in a triple negative (ER, PgR, HER2-negative) group. For patients with 'vimentin or CK5/6, 14, 17-positive' tumours, 5-year estimated survival rate was 78.6%, whereas for patients with 'vimentin, or CK5/6, 14, 17-negative' tumours it was 58.3% (log-rank p = 0.227).

**Conclusion:**

We were not able to better delineate an immunohistochemical definition of basal type of breast cancer by adding vimentin to the immunopanel consisted of ER, PgR, HER2, CK5/6, 14 and 17 markers, when overall survival was a primary end-point.

## Background

Vimentin is a 57 kDa intermediate filament (IF) protein, which forms a part of the cytoskeleton. Six major classes of IFs are believed to be relatively specific for certain cell types, for example keratin in epithelial cells, neurofilaments in neurons, glial fibrillary acid protein in glial cells, desmin in muscule cells and vimentin in mesenchymal cells. Obviously, they are variably expressed in different cell types and in corresponding tumours. Expression of vimentin and cytokeratins has also been described in breast carcinomas [1,2].

Moreover, vimentin is selectively expressed in aggressive breast cancer cell lines [3]. Elevated vimentin expression level correlates well with up-regulated migration and invasion of cancer cells [3,4]. The transfection of the non-invasive human breast cancer cell line (MCF7) with vimentin gene led to accelerated invasiveness [5]. Other data showed that more invasive breast cancer lines expressed vimentin, suggesting its usefulness in identifying cases with poorer prognosis [6].

Vimentin reactive cells in benign and malignant breast tissue have been described by many authors [4,7]. The same applies to a possible association with clinically aggressive behavior of tumours [7], which may be explained by correlation with estrogen receptor negativity [8,9], high Ki-67 level [9] and poor differentiation of tumours (high grade) [10,11].

Few reports are in opposite, as they showed that vimentin expression did not inversely predict patient survival [12].

The cDNA microrray experiments enabled the identification of different subgroups of breast tumours with distinct molecular signatures [13-15]. This molecular classification delineated at least four biologically different phenotypes: luminal phenotype (generally, estrogen receptor positive tumours), normal breast-like phenotype and estrogen receptor negative tumours, comprising the subgroups of HER2 (overexpression of *ERBB2 *oncogene) and basal-like phenotypes (tumours expressing genetic markers that are characteristic of the myoepithelium of the normal mammary gland, such as epidermal growth factor receptor, p63 and basal cytokeratins CK 5/6, CK 14, CK17 [13-15]. It is also known that a subgroup with HER 2 overexpression and basal-like phenotype correlate with poor prognosis. Many efforts have been undertaken to reproduce this classification with the use of immunohistochemistry instead of assessment of mRNA [16-18].

Some researchers suggested that immunohistochemically triple negative tumours (ER, PgR, and HER 2-negativity) could reliably be defined as basal-like tumours, making these two subgroups synonymous [19]. Others believe that equating triple negative tumours with basal-like breast cancer is misleading [20]. However, there is a common agreement that the key point of basal-like characteristics is triple negativity of tumours. On the other hand, it should be stressed that not only basal-like cancers harbour a triple negative phenotype at the mRNA level, and normal-breast like cancers also have this feature [13,21]. It has been shown that typical features of basal-like tumours include the expression of: high molecular weight cytokeratins – CK5/6, 14, 17 (so-called basal type cytokeratins) [18,22,23], expression of epidermal growth factor receptor (EGFR), c-kit, P53, and vimentin [4,16,18,20,23,24].

Recent studies have compared the prognostic significance of three-(ER, PgR, HER2) and five-biomarker (ER, PgR, HER2, CK5/6, EGFR) surrogate panels to define intrinsic breast cancer subtypes and have suggested that the extended immunopanel provided more specific definition of basal like breast cancer, which can better predict survival of breast cancer patients [25].

The aim of our study was to assess if the immunopanel consisted of triple negative phenotype (ER, PgR, HER2) with the addition of basal cytokeratins (CK5/6, 14, 17) or vimentin could better delineate a basal type tumour group and better predict patient survival when compared to only pure ER, PgR, HER2 negative phenotype.

## Materials and methods

A series of 179 formalin fixed, paraffin-embedded invasive ductal carcinomas not otherwise specified were acquired from the archives of the Pathology Department of Copernicus Memorial Hospital, Lodz, Poland. Patients had undergone surgery (total mastectomy with axillary lymph node dissection) between 1997 and 2001. The median patient age at surgery was 56 years (range, 25–92 years).

The primary pathologic diagnosis was confirmed in H&E staining. All operative and pathologic reports were reviewed to confirm disease stage. Follow-up period was defined as a time from surgery to the last observation for censored cases or death for complete observations.

### Immunohistochemistry and scoring

Sections of 2 μm thickness were cut and mounted onto polylysine-coated slides, which were stained for vimentin, estrogen receptor (ER), progesterone receptor (PgR), HER2, cytokeratin 5/6, 14 and 17, Ki-67, cyclin E and p-cadherin.

### Staining procedures

Antibodies against:

- vimentin (Dako), dilution 1:50, antigen retrieval: autoclave, high pH;

- CK5/6 (Dako), 1:100, autoclave, high pH;

- CK 14 (Novocastra), 1:20, microwave oven, citrate buffer, pH 6;

- CK17 (Novocastra), 1:40, microwave oven, citrate buffer, pH 6;

- ER (Dako),1:35, microwave oven, citrate buffer, pH 6;

- PgR (Dako),1:75, microwave oven, citrate buffer, pH 6;

- HER2 (Herceptest, Dako) and Ki-67 (Dako), 1:200, microwave oven, citrate buffer, pH 6;

- cyclin E (Dako), 1:40, microwave oven, citrate buffer, pH 6;

- p-cadherin (Dako), 1:200, microwave oven, citrate buffer, pH 6.

### Scoring

Any distinct positive staining of tumour parenchyma with vimentin antibody was regarded as vimentin expression. Positive staining in fibroblasts, endothelial cells, lymphocytes and macrophages served as 'built-in' positive control, furthermore, negative staining of epithelial cells in non-neoplastic tubules served as negative control.

For CK5/6, CK14 and CK17, membranous staining results were classified as follows: negative – no staining seen in invasive tumour cells, positive – weak or strong staining seen in invasive cancer cells.

ER and PgR nuclear staining scoring was done using the method described by McCarty et al. [26]. Tumours were considered as being positive for ER or PgR if Histo-score was above 100.

HER2 staining was scored according to Herceptest kit manufacturer's instructions and score 3+ denoted HER2-positive tumours.

Ki-67 and cyclin E labeling indices were defined as the percentages of tumour cells displaying nuclear immunoreactivity and were calculated by counting nuclear stained tumour cells in 1000 tumour cells. For cyclin E, samples were classified as being negative (<2%) or positive (≥ 2%).

For p-cadherin, a semiquantitative scoring system was used, taking into account both the intensity of staining and the proportion of tumour cells showing the positive reaction. The scores of staining intensity were recorded from 0 (no staining) to 3 (strong staining). The scores of staining area were recorded as 1 (<10%), 2 (10–50%) or 3 (>50%). A staining index (SI) was obtained by multiplying the score of staining intensity by the score of staining area, negative cases had SI = 0–1, positive ones had SI = 2–9.

### Statistical analysis

Pearson's chi-square test or Fisher exact test were used to test for contingency between dichotomized values of vimentin expression (negative and positive) and values of other histopathological and clinical parameters. Patient survival was calculated from the date of primary surgery to the date of death or the last follow-up according to the Kaplan-Meier method. Data for patients who died from other causes than breast cancer were censored at the time of death. Differences in survival distributions were evaluated by a log-rank test. Univariate survival analyses were performed with the use of Cox proportional hazards method. All results were considered statistically significant when two-sided p was less than 0.05. The analyses were performed using the StatsDirect software (StatsDirect Ltd., UK).

## Results

### Patient characteristics and vimentin expression

The median follow-up period for all patients was 71 months (range, 1–130), and for 113 censored (living) patients it was 90 months (range, 9–130).

**Figure 1:**
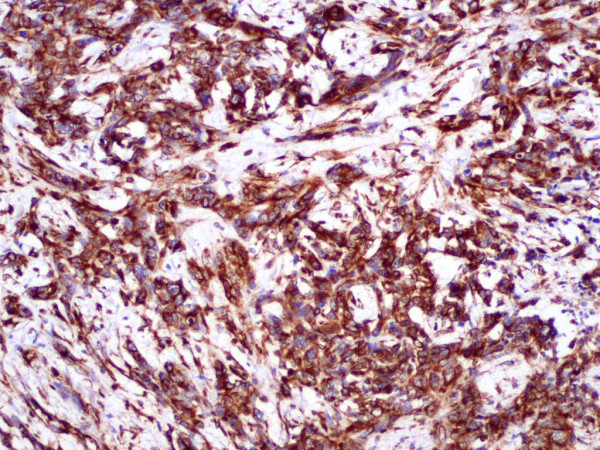
**Positive staining for vimentin**. Breast cancer, magnification × 100.

Vimentin expression was observed in 38 cases (21.2%) (Table 1, Fig. 1), whereas 141 (78.8%) (Table 1) tumours were found to be vimentin-negative.

**Table 1:** Associations between clinical and histopathological features and expression of vimentin.

Feature	Vimentin-negative N = 141	Vimentin-positive N = 38	p value
Age (mean)	58.09	51.79	0.024

Tumour size			0.294
T1	43	15	
T2-4	98	23	

Nodal status			0.718
Negative	64	16	
Positive	77	22	

Grading			<0.001
G1-2	90	10	
G3	51	28	

ER			<0.001
Negative	70	31	
Positive	71	7	

PgR			<0.001
Negative	64	31	
Positive	77	7	

CK5/6			<0.001
Negative	109	8	
Positive	32	30	

CK14			<0.001
Negative	134	21	
Positive	7	17	

CK17			<0.001
Negative	118	16	
Positive	23	22	

CK5/6 or 14 o r17			<0.001
Negative	105	8	
Positive	36	30	

HER2			0.004
Negative	110	37	
Positive	31	1	

Triple negativity			<.001
Yes	25	29	
No	116	9	

P-cadherin			0.110
Negative	61	11	
Positive	80	27	

Cyclin E			0.058
Negative	65	11	
Positive	76	27	

Ki-67 expression, % (mean)	9.09	11.34	0.152

The second and third columns contain numbers of patients, age and Ki-67 expression excepted.

Women with vimentin-positive cancers were significantly younger when compared with vimentin-negative ones (Table 1).

Among 38 vimentin-positive tumours, 31 were ER-negative and 31 were PgR-negative, whereas 7 were ER and PgR-positive (p < 0.001) (Table 1). Also 37 cases were HER2-negative and only 1 was positive (p < 0.004) (Table 1). Moreover, vimentin expressing tumours were usually positive for at least one of the basal type cytokeratins (CK5/6 or CK14 or CK17) (p < 0.001) (Table 1).

Vimentin-positive tumours were significantly more often high grade tumours. Such relationship was very strong in all patients (p < 0.001) and significant in triple negative tumours (p = 0.035). In the non-triple negative group only not significant tendency towards such relationship was observed (p = 0.065).

There was also a statistically insignificant but quite obvious tendency towards a relationship between vimentin and cyclin E. Vimentin-positive tumours more frequently expressed cyclin E (p = 0.058) (Table 1). Relation with Ki-67 and p-cadherin did not attain statistical significance (p = 0.152 and p = 0.110, respectively) (Table 1).

54 patients had triple negative tumours (30.2%) (Table 2), whereas non-triple negative phenotype defined as the expression of at least one of the three markers (ER, PgR or HER2) was observed in 125 patients (69.8%) (Table 2). Among 54 triple negative tumours, 39 (72.2%) were 'CK5/6 or 14 or 17'-positive and 15 (27.8%) were negative for these keratins. 'Vimentin or CK5/6 or 14 or 17' positivity was established for 42 (77.8%), and negativity for 12 (22.2%) triple negative tumours.

**Table 2:** Prognostic value of basal type breast cancer delineated by two different immunopanels.

Subgroup	Hazard ratio (95%CI)	p value	5-year survival rate (95%CI) (%)	p value (log-rank)
All patients (n = 179)				

'CK5/6 or 14 or 17'	1.46 (0.90–2.37)	0.127		0.124
Positive			63.5 (50.7–73.8)	
Negative			75.3 (66.1–82.4)	

Vimentin	1.22 (0.69–2.14)	0.497		0.496
Positive			59.5 (42.1–73.3)	
Negative			73.9 (65.7–80.4)	

'Vimentin or CK5/6 or	1.73 (1.07–2.81)	0.026		0.024
14 or 17'				
Positive			61.5 (49.3–71.6)	
Negative			77.6 (68.2–84.5)	

Triple negative patients (n = 54)				

'CK5/6 or 14 or 17'	0.50 (0.21–1.20)	0.122		0.115
Positive			71.8 (54.9–83.3)	
Negative			52.5 (25.2–74.0)	

Vimentin	0.64 (0.28–1.48)	0.297		0.293
Positive			69.0 (48.8–82.5)	
Negative			68.0 (46.1–82.5)	

'Vimentin or CK5/6 or	0.56 (0.22–1.45)	0.234		0.227
14 or 17'				
Positive			78.6 (62.9–88.2)	
Negative			58.3 (27.0–80.1)	

Non-triple negative patients (n = 125)				

'CK5/6 or 14 or 17'	2.61 (1.40–4.84)	0.002		0.002
Positive			50.9 (30.7–67.9)	
Negative			77.8 (67.9–84.9)	

Vimentin*	3.26 (1.37–7.77)	0.008		0.005
Positive			25.4 (3.8–56.4)	
Negative			75.2 (66.1–82.2)	

'Vimentin or CK5/6 or	3.04 (1.66–5.56)	<0.001		<0.001
14 or 17'				
Positive			47.5 (29.1–63.8)	
Negative			80.1 (70.2–87.0)	

*In a non-triple negative group only 9 patients were positive for vimentin. Thus, results of survival analysis should be regarded as being inconclusive and they are showed for comparative purposes only.

### Survival analysis

#### All (n = 179) patients

**Figure 2:**
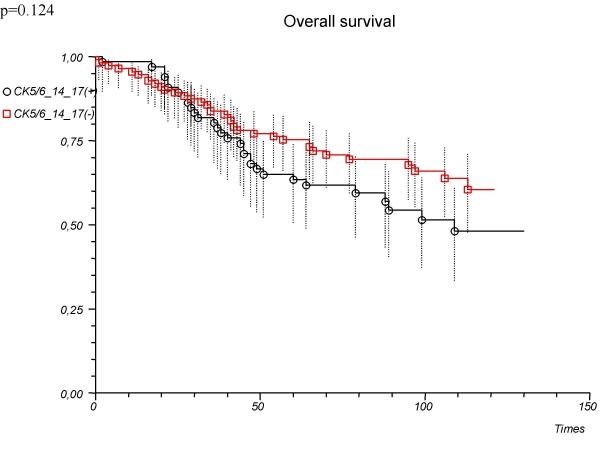
**Overall survival depending on the immunopanel ('CK5/6 or 14 or 17') used in the determination of basal type tumours**. All patients (n = 179).

**Figure 3:**
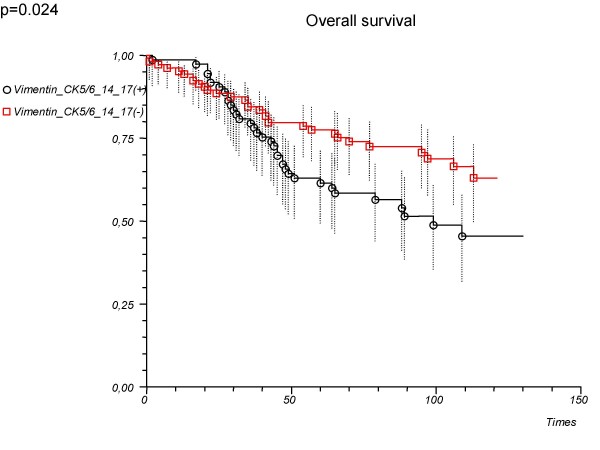
**Overall survival depending on the immunopanel ('Vimentin or CK5/6 or 14 or 17') used in the determination of basal type tumours**. All patients (n = 179).

As a single marker, vimentin was not associated significantly with patient survival (hazard ratio 1.22, 95%CI 0.69–2.14, p = 0.497; log-rank p = 0.496) (Table 2). Also compilation of basal cytokeratins (CK5/6 or CK14 or CK17 – positive vs. negative tumours) was not associated significantly with patient survival (hazard ratio 1.46, 95%CI 0.90–2.37, p = 0.127; log-rank p = 0.124) (Table 2, Fig. 2). However, adding vimentin to basal cytokeratins compilation (vimentin or CK5/6 or CK14 or CK17-positive vs. negative tumours) could significantly determine the prognosis (Table 2, Fig. 3).

#### Patients with triple negative tumours (n = 54)

In 54 (30.2%) triple negative patients vimentin as a single marker did not predict clinical outcome (hazard ratio 0.64, 95%CI 0.28–1.48, p = 0.297; log-rank p = 0.293) (Table 2).

**Figure 4:**
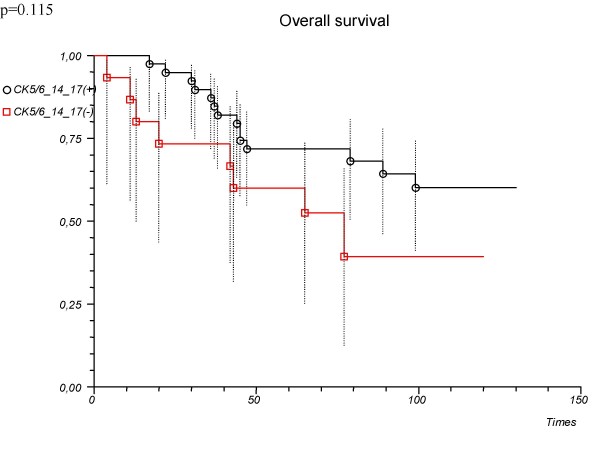
**Overall survival depending on the immunopanel ('CK5/6 or 14 or 17') used in the determination of basal type tumours**. Patients with triple negative cancer (n = 54).

**Figure 5:**
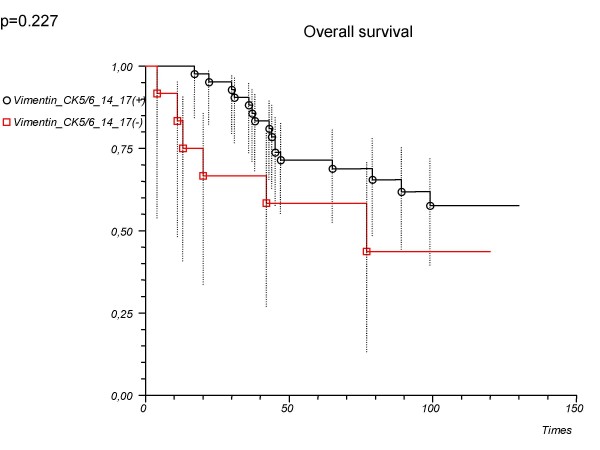
**Overall survival depending on the immunopanel ('Vimentin or CK5/6 or 14 or 17') used in the determination of basal type tumours**. Patients with triple negative cancer (n = 54).

There was a tendency towards slightly better outcome in 'CK5/6 or 14 or 17'-positive patients when compared with the negative ones but this difference was not significant (Table 2, Fig. 4). There was no significant difference in clinical outcome between 'vimentin or CK5/6 or 14 or 17' – positive vs. negative patients (Table 2, Fig. 5).

#### Patients with non-triple negative tumours (n = 125)

In a non-triple negative group only 9 patients were positive for vimentin. Thus, results of survival analysis shown in Table 2 should be regarded as being inconclusive and they are presented for comparative purposes only.

## Discussion

In this study, positive staining for vimentin was found in 21.2% of cases, the proportion which is similar [9], smaller [12] or higher [2] to reported by others. Such disagreements between studies could be possibly explained by the subjectivity of the method and differences between scoring systems used. Some authors have pointed out that differences in vimentin expression may depend on the type of tissue fixation – the smaller amount of vimentin-expressing cells is observed in formalin fixed, paraffin-embedded tissues [27,28]. In our study, there was a statistically significant correlation between vimentin expression and poor differentiation of tumours (G3 cancers) both in all patients and in the triple negative group. In the non-triple negative group, due to the limited number of vimentin-positive tumours, only not significant, although an obvious tendency towards such relationship was observed. Our observations remain in concordance with data published by others [10,29,30]. Also, vimentin expressing tumours had slightly higher Ki-67 level, but without statistical significance, so this particular result is not supported by other analyses [4,9]. Published data showed significant associations between basal keratins expression (CK5/6, CK14) and vimentin expression [23]. In our study, a very strong (p < 0.001) association between vimentin expression and expression of at least one of the basal type cytokeratin (CK5/6 or CK14 or CK17) was also confirmed. In the present study, vimentin-positive cancers were more often found in younger women. This result remains to some extent in contrast with observations made by Chen at al. that vimentin and basal cytokeratins were expressed at significantly lower lewels in breast cancer cells from women aged 31 years and below compared with those from patients between 32 and 35 years old [30]. However, Abd El-Rehim at al. and Cheang at al. have found correlation between basal markers expression and younger patient age [18,25].

Univariate survival analysis, for all patients, showed that vimentin expression did not influence the clinical outcome, so we agree with some researchers who have shown that vimentin positivity was not associated with any difference in patient survival [12,29]. Thus, we cannot support the hypothesis suggesting the usefulness of vimentin as a single marker in identifying cases with poorer prognosis [9]. Only in the group of non-triple negative patients, vimentin expression attains significance with survival of patients (p = 0.005) but this group contains only 9 positive cases, so we consider this results as being inconclusive and we have showed them for comparative purposes only.

In our study, an immunopanel containing 'vimentin-positive or basal cytokeratin (CK5/6, 14, 17)-positive and triple negative (ER, PGR, HER2)' markers was formulated and its prognostic value has been checked out by the comparison with 'basal cytokeratin (CK5/6, 14, 17)-positive and triple negative (ER, PGR, HER2)' panel, in which vimentin is omitted.

These two basal phenotype immunopanels were adversely associated with survival in patients with non-triple negative cancer (Table 2). This effect was far less evident in a group of all patients – only a four-marker immunopanel consisting of CK5/6, CK14, CK17 and vimentin was significantly related to the clinical outcome. This can be explained at least partially by correlation of vimentin expression with ER and PgR negativity, and with higher grade of cancer. However, the main purpose of the present study was to assess the prognostic usefulness of basal markers including vimentin in a triple negative group. We have found that in triple negative patients none of the basal phenotype immunopanels was adversely related with patients' survival. On the contrary, there was an insignificant tendency towards better prognosis when basal keratins or vimentin were detected in a primary tumour. This observation remains to some extent in contrast with observations made by Cheang et al. [25], Liu et al. [31], and by Rakha et al. [32]. However, Jumppanen et al. have found that the clinical outcome of basal tumours is similar to non-basal ER-negative tumours [33]. Moreover, they have observed that basal keratins expression significantly affected survival only during the first 5 years of follow-up and lost its significance later on. In our study the median follow-up period in a group of surviving patients was 7.5 years and our observation corresponds well with observations made by Jumppanen and colleagues [33]. Indeed, Tischkowitz et al. have found that the difference in survival rate between triple negative and non-triple negative breast cancer is reduced with longer follow-up period [34]. When basal phenotype markers like CK 5/6 and HER1 (EGFR) were analyzed without consideration of steroid receptors status, the reduction in survival of patients expressing these markers was more pronounced at 10 years of observation that at 3 years. Our results, although restricted by a relative small number of patients with triple negative phenotype, confirm these findings.

The present study also supports our previous analysis which showed that basal cytokeratins (CK5/6 and CK17) expression had not any impact on survival in patients with breast cancer [35].

The possible association of vimentin with clinically aggressive behaviour of tumours described by others [7-9,11] may be explained by the correlation of vimentin expression with lack of steroid receptors and poor differentiation of cancer. We can confirm this observation (Table 1).

However, we cannot offer a better indicator of basal type breast cancers by adding vimentin to the diagnostic panel when overall survival is a primary end-point. Also, an immunopanel defined as CK5/6 or 14 or 17-positivity did not show any significant prognostic value in survival analysis in a triple negative group. Five marker method proposed by Cheang et al. [25] showed superior prognostic value than only triple negative phenotype. In their analysis, triple negative, CK5/6-positive and EGFR-positive tumours were selected. Taken into consideration a strong positive correlation between EGFR and vimentin expression [4], we have taken an effort to construct an immunopanel defining basal-type tumours as triple negative tumours that are vimentin-positive or basal cytokeratin-positive. In a comparison with Cheang's study, our analysis was based on a smaller number of patients and instead of EGFR, vimentin expression was applied. However, in our study, the median follow-up period in a group of living patients almost reached 8 years. To understand, why the presence of vimentin or basal cytokeratins is not related to triple negative patient outcome, further studies should be undertaken.

## Conclusion

In summary, may we conclude that adding vimentin to an immunopanel consisted of basal cytokeratins (CK5/6, 14, 17) appears to be inefficient at predicting survival of triple negative breast cancer patients.

## Competing interests

The authors declare that they have no competing interests.

## Authors' contributions

RUK – conceived and coordinated the study, performed experiments, analyses, interpreted data and wrote the manuscript. RK – acquisition of funding, general supervision of the research group. EP, AKB, JHP – acquisition of data, edition of the draft manuscript. PP – participated in analysis and interpretation of data, performed the statistical analysis, was involved in drafting the manuscript and revised it critically. All authors read and approved the final manuscript.
